# Impact of intensive prone position therapy on outcomes in intubated patients with ARDS related to COVID-19

**DOI:** 10.1186/s13613-024-01340-z

**Published:** 2024-06-27

**Authors:** Christophe Le Terrier, Thaïs Walter, Said Lebbah, David Hajage, Florian Sigaud, Claude Guérin, Luc Desmedt, Steve Primmaz, Vincent Joussellin, Chiara Della Badia, Jean-Damien Ricard, Jérôme Pugin, Nicolas Terzi, COVID-ICU Group on behalf of the REVA Network and the COVID-ICU Investigators, Christophe Le Terrier, Said Lebbah, David Hajage, Steve Primmaz, Jean-Damien Ricard, Jérôme Pugin, Nicolas Terzi, Alain Mercat, Pierre Asfar, François Beloncle, Julien Demiselle, Tài Pham, Arthur Pavot, Xavier Monnet, Christian Richard, Alexandre Demoule, Martin Dres, Julien Mayaux, Alexandra Beurton, Cédric Daubin, Richard Descamps, Aurélie Joret, Damien Du Cheyron, Frédéric Pene, Jean-Daniel Chiche, Mathieu Jozwiak, Paul Jaubert, Guillaume Voiriot, Muriel Fartoukh, Marion Teulier, Clarisse Blayau, Erwen L’Her, Cécile Aubron, Laetitia Bodenes, Nicolas Ferriere, Johann Auchabie, Anthony Le Meur, Sylvain Pignal, Thierry Mazzoni, Jean-Pierre Quenot, Pascal Andreu, Jean-Baptiste Roudau, Marie Labruyère, Saad Nseir, Sébastien Preau, Julien Poissy, Daniel Mathieu, Sarah Benhamida, Rémi Paulet, Nicolas Roucaud, Martial Thyrault, Florence Daviet, Sami Hraiech, Gabriel Parzy, Aude Sylvestre, Sébastien Jochmans, Anne-Laure Bouilland, Mehran Monchi, Marc Danguy des Déserts, Quentin Mathais, Gwendoline Rager, Pierre Pasquier, Reignier Jean, Seguin Amélie, Garret Charlotte, Canet Emmanuel, Jean Dellamonica, Clément Saccheri, Romain Lombardi, Yanis Kouchit, Sophie Jacquier, Armelle Mathonnet, Mai-Ahn Nay, Isabelle Runge, Frédéric Martino, Laure Flurin, Amélie Rolle, Michel Carles, Rémi Coudroy, Arnaud W. Thille, Jean-Pierre Frat, Maeva Rodriguez, Pascal Beuret, Audrey Tientcheu, Arthur Vincent, Florian Michelin, Fabienne Tamion, Dorothée Carpentier, Déborah Boyer, Christophe Girault, Valérie Gissot, Stéphan Ehrmann, Charlotte Salmon Gandonniere, Djlali Elaroussi, Agathe Delbove, Yannick Fedun, Julien Huntzinger, Eddy Lebas, Grâce Kisoka, Céline Grégoire, Stella Marchetta, Bernard Lambermont, Laurent Argaud, Thomas Baudry, Pierre-Jean Bertrand, Auguste Dargent, Christophe Guitton, Nicolas Chudeau, Mickaël Landais, Cédric Darreau, Alexis Ferre, Antoine Gros, Guillaume Lacave, Fabrice Bruneel, Mathilde Neuville, Jérôme Devaquet, Guillaume Tachon, Richard Gallot, Riad Chelha, Arnaud Galbois, Anne Jallot, Ludivine Chalumeau Lemoine, Khaldoun Kuteifan, Valentin Pointurier, Louise-Marie Jandeaux, Joy Mootien, Charles Damoisel, Benjamin Sztrymf, Matthieu Schmidt, Alain Combes, Juliette Chommeloux, Charles Edouard Luyt, Frédérique Schortgen, Leon Rusel, Camille Jung, Florent Gobert, Damien Vimpere, Lionel Lamhaut, Bertrand Sauneuf, Liliane Charrrier, Julien Calus, Isabelle Desmeules, Benoît Painvin, Jean-Marc Tadie, Vincent Castelain, Baptiste Michard, Jean-Etienne Herbrecht, Mathieu Baldacini, Nicolas Weiss, Sophie Demeret, Clémence Marois, Benjamin Rohaut, Pierre-Henri Moury, Anne-Charlotte Savida, Emmanuel Couadau, Mathieu Série, Nica Alexandru, Cédric Bruel, Candice Fontaine, Sonia Garrigou, Juliette Courtiade Mahler, Maxime Leclerc, Michel Ramakers, Pierre Garçon, Nicole Massou, Ly Van Vong, Juliane Sen, Nolwenn Lucas, Franck Chemouni, Annabelle Stoclin, Alexandre Avenel, Henri Faure, Angélie Gentilhomme, Sylvie Ricome, Paul Abraham, Céline Monard, Julien Textoris, Thomas Rimmele, Florent Montini, Gabriel Lejour, Thierry Lazard, Isabelle Etienney, Younes Kerroumi, Claire Dupuis, Marine Bereiziat, Elisabeth Coupez, François Thouy, Clément Hoffmann, Nicolas Donat, Anne Chrisment, Rose-Marie Blot, Antoine Kimmoun, Audrey Jacquot, Matthieu Mattei, Bruno Levy, Ramin Ravan, Loïc Dopeux, Jean-Mathias Liteaudon, Delphine Roux, Brice Rey, Radu Anghel, Deborah Schenesse, Vincent Gevrey, Jermy Castanera, Philippe Petua, Benjamin Madeux, Otto Hartman, Michael Piagnerelli, Anne Joosten, Cinderella Noel, Patrick Biston, Thibaut Noel, Gurvan Le Bouar, Messabi Boukhanza, Elsa Demarest, Marie-France Bajolet, Nathanaël Charrier, Audrey Quenet, Cécile Zylberfajn, Nicolas Dufour, Buno Mégarbane, Sqébastian Voicu, Nicolas Deye, Isabelle Malissin, François Legay, Matthieu Debarre, Nicolas Barbarot, Pierre Fillatre, Bertrand Delord, Thomas Laterrade, Tahar Saghi, Wilfried Pujol, Pierre Julien Cungi, Pierre Esnault, Mickael Cardinale, Vivien Hong Tuan Ha, Grégory Fleury, Marie-Ange Brou, Daniel Zafimahazo, David Tran-Van, Patrick Avargues, Lisa Carenco, Nicolas Robin, Alexandre Ouali, Lucie Houdou, Noémie Suh, Emmanuel Weiss, Tobias Gauss, Jean-Denis Moyer, Catherine Paugam Burtz, Béatrice La Combe, Rolland Smonig, Jade Violleau, Pauline Cailliez, Antoine Marchalot, Cécile Saladin, Christelle Bigot, Pierre-Marie Fayolle, Jules Fatséas, Amr Ibrahim, Dabor Resiere, Rabih Hage, Clémentine Cholet, Marie Cantier, Pierre Trouiler, Philippe Montravers, Brice Lortat-Jacob, Sebastien Tanaka, Alexy Tran Dinh, Jacques Duranteau, Anatole Harrois, Guillaume Dubreuil, Marie Werner, Anne Godier, Sophie Hamada, Diane Zlotnik, Hélène Nougue, Armand Mekontso-Dessap, Guillaume Carteaux, Keyvan Razazi, Nicolas De Prost, Nicolas Mongardon, Olivier Langeron, Eric Levesque, Arié Attias, Charles de Roquetaillade, Benjamin G. Chousterman, Alexandre Mebazaa, Etienne Gayat, Marc Garnier, Emmanuel Pardo, Lea Satre-Buisson, Christophe Gutton, Elise Yvin, Clémence Marcault, Elie Azoulay, Michael Darmon, Hafid Ait Oufella, Geoffroy Hariri, Tomas Urbina, Sandie Mazerand, Nicholas Heming, Francesca Santi, Pierre Moine, Djillali Annane, Adrien Bouglé, Edris Omar, Aymeric Lancelot, Emmanuelle Begot, Gaétan Plantefeve, Damien Contou, Hervé Mentec, Olivier Pajot, Stanislas Faguer, Olivier Cointault, Laurence Lavayssiere, Marie-Béatrice Nogier, Matthieu Jamme, Claire Pichereau, Jan Hayon, Hervé Outin, François Dépret, Maxime Coutrot, Maité Chaussard, Lucie Guillemet, Pierre Goffin, Romain Thouny, Julien Guntz, Laurent Jadot, Romain Persichini, Vanessa Jean-Michel, Hugues Georges, Thomas Caulier, Gaël Pradel, Marie-Hélène Hausermann, Thi My Hue Nguyen-Valat, Michel Boudinaud, Emmanuel Vivier, Sylvène Rosseli, Gaël Bourdin, Christian Pommier, Marc Vinclair, Simon Poignant, Sandrine Mons, Wulfran Bougouin, Franklin Bruna, Quentin Maestraggi, Christian Roth, Laurent Bitker, François Dhelft, Justine Bonnet-Chateau, Mathilde Filippelli, Tristan Morichau-Beauchant, Stéphane Thierry, Charlotte Le Roy, Mélanie Saint Jouan, Bruno Goncalves, Aurélien Mazeraud, Matthieu Daniel, Tarek Sharshar, Cyril Cadoz, Rostane Gaci, Sébastien Gette, Guillaune Louis, Sophe-Caroline Sacleux, Marie-Amélie Ordan, Aurélie Cravoisy, Marie Conrad, Guilhem Courte, Sébastien Gibot, Younès Benzidi, Claudia Casella, Laurent Serpin, Jean-Lou Setti, Marie-Catherine Besse, Anna Bourreau, Jérôme Pillot, Caroline Rivera, Camille Vinclair, Marie-Aline Robaux, Chloé Achino, Marie-Charlotte Delignette, Tessa Mazard, Frédéric Aubrun, Bruno Bouchet, Aurélien Frérou, Laura Muller, Charlotte Quentin, Samuel Degoul, Xavier Stihle, Claude Sumian, Nicoletta Bergero, Bernard Lanaspre, Hervé Quintard, Eve Marie Maiziere, Pierre-Yves Egreteau, Guillaume Leloup, Florin Berteau, Marjolaine Cottrel, Marie Bouteloup, Matthieu Jeannot, Quentin Blanc, Julien Saison, Isabelle Geneau, Romaric Grenot, Abdel Ouchike, Pascal Hazera, Anne-Lyse Masse, Suela Demiri, Corinne Vezinet, Elodie Baron, Deborah Benchetrit, Antoine Monsel, Grégoire Trebbia, Emmanuelle Schaack, Raphaël Lepecq, Mathieu Bobet, Christophe Vinsonneau, Thibault Dekeyser, Quentin Delforge, Imen Rahmani, Bérengère Vivet, Jonathan Paillot, Lucie Hierle, Claire Chaignat, Sarah Valette, Benoït Her, Jennifier Brunet, Mathieu Page, Fabienne Boiste, Anthony Collin, Florent Bavozet, Aude Garin, Mohamed Dlala, Kais Mhamdi, Bassem Beilouny, Alexandra Lavalard, Severine Perez, Benoit Veber, Pierre-Gildas Guitard, Philippe Gouin, Anna Lamacz, Fabienne Plouvier, Bertrand P. Delaborde, Aïssa Kherchache, Amina Chaalal, Marc Amouretti, Santiago Freita-Ramos, Damien Roux, Jean-Michel Constantin, Mona Assefi, Marine Lecore, Agathe Selves, Florian Prevost, Christian Lamer, Ruiying Shi, Lyes Knani, Sébastien Pili Floury, Lucie Vettoretti, Michael Levy, Lucile Marsac, Stéphane Dauger, Sophie Guilmin-Crépon, Hadrien Winiszewski, Gael Piton, Thibaud Soumagne, Gilles Capellier, Jean-Baptiste Putegnat, Frédérique Bayle, Maya Perrou, Ghyslaine Thao, Guillaume Géri, Cyril Charron, Xavier Repessé, Antoine Vieillard-Baron, Mathieu Guilbart, Pierre-Alexandre Roger, Sébastien Hinard, Pierre-Yves Macq, Kevin Chaulier, Sylvie Goutte, Patrick Chillet, Anaïs Pitta, Barbara Darjent, Amandine Bruneau, Sigismond Lasocki, Maxime Leger, Soizic Gergaud, Pierre Lemarie, Carole Schwebel, Anaïs Dartevel, Louis-Marie Galerneau, Jean-Luc Diehl, Caroline Hauw-Berlemont, Nicolas Péron, Emmanuel Guérot, Abolfazl Mohebbi Amoli, Michel Benhamou, Jean-Pierre Deyme, Olivier Andremont, Diane Lena, Julien Cady, Arnaud Causeret, Arnaud De La Chapelle, Christophe Cracco, Stéphane Rouleau, David Schnell, Camille Foucault, Cécile Lory, Thibault Chapelle, Vincent Bruckert, Julie Garcia, Abdlazize Sahraoui, Nathalie Abbosh, Caroline Bornstain, Pierre Pernet, Florent Poirson, Ahmed Pasem, Philippe Karoubi, Virginie Poupinel, Caroline Gauthier, François Bouniol, Philippe Feuchere, Anne Heron, Serge Carreira, Malo Emery, Anne Sophie Le Floch, Luana Giovannangeli, Nicolas Herzog, Christophe Giacardi, Thibaut Baudic, Chloé Thill, Jessica Palmyre, Florence Tubach, Nicolas Bonnet, Nathan Ebstein, Stéphane Gaudry, Yves Cohen, Julie Noublanche, Olivier Lesieur, Arnaud Sément, Isabel Roca-Cerezo, Michel Pascal, Nesrine Sma, Gwenhaël Colin, Jean-Claude Lacherade, Gauthier Bionz, Natacha Maquigneau, Pierre Bouzat, Michel Durand, Marie-Christine Hérault, Jean-Francois Payen

**Affiliations:** 1 Division of Intensive Care, Faculty of Medicine, Geneva University Hospitals and the University of Geneva, Gabrielle-Perret-Gentil 4, 1205 Geneva, Switzerland; 2 Division of Intensive Care, Saint-Louis Hospital, Greater Paris Hospital, Paris, France; 3 Département de Santé Publique, Centre de Pharmaco-épidémiologie, AP-HP, Paris, France; 4 Division of Intensive Care, Grenoble Alpes University Hospital, Grenoble, France; 5 Division of Intensive Care, Edouard Herriot University Hospital, Lyon, France; 6 Medical Intensive Care Unit, Nantes Hôtel-Dieu University Hospital, Nantes, France; 7 Medical Intensive Care Unit, University Hospital of Rennes, Rennes, France; 8 UMR1137 IAME, INSERM, Université Paris Cité, 75018 Paris, France; 9 DMU ESPRIT, Service de Médecine Intensive Réanimation, Université Paris Cité, AP-HP, Hôpital Louis Mourier, 92700 Colombes, France

**Keywords:** Acute respiratory distress syndrome, Intubation, COVID-19, Mortality, Intensive prone position, Intensive care unit

## Abstract

**Background:**

Previous retrospective research has shown that maintaining prone positioning (PP) for an average of 40 h is associated with an increase of survival rates in intubated patients with COVID-19-related acute respiratory distress syndrome (ARDS). This study aims to determine whether a cumulative PP duration of more than 32 h during the first 2 days of intensive care unit (ICU) admission is associated with increased survival compared to a cumulative PP duration of 32 h or less.

**Methods:**

This study is an ancillary analysis from a previous large international observational study involving intubated patients placed in PP in the first 48 h of ICU admission in 149 ICUs across France, Belgium and Switzerland. Given that PP is recommended for a 16-h daily duration, intensive PP was defined as a cumulated duration of more than 32 h during the first 48 h, whereas standard PP was defined as a duration equal to or less than 32 h. Patients were followed-up for 90 days. The primary outcome was mortality at day 60. An Inverse Probability Censoring Weighting (IPCW) Cox model including a target emulation trial method was used to analyze the data.

**Results:**

Out of 2137 intubated patients, 753 were placed in PP during the first 48 h of ICU admission. The intensive PP group (*n* = 79) had a median PP duration of 36 h, while standard PP group (*n* = 674) had a median of 16 h during the first 48 h. Sixty-day mortality rate in the intensive PP group was 39.2% compared to 38.7% in the standard PP group (*p* = 0.93). Twenty-eight-day and 90-day mortality as well as the ventilator-free days until day 28 were similar in both groups. After IPCW, there was no significant difference in mortality at day 60 between the two-study groups (HR 0.95 [0.52–1.74], *p* = 0.87 and HR 1.1 [0.77–1.57], *p* = 0.61 in complete case analysis or in multiple imputation analysis, respectively).

**Conclusions:**

This secondary analysis of a large multicenter European cohort of intubated patients with ARDS due to COVID-19 found that intensive PP during the first 48 h did not provide a survival benefit compared to standard PP.

**Supplementary Information:**

The online version contains supplementary material available at 10.1186/s13613-024-01340-z.

## Introduction

Prone positioning (PP) for intubated patients with acute respiratory distress syndrome (ARDS) has been widely implemented during the COVID-19 pandemic [1]. In large cohorts of mechanically ventilated patients, approximately 70% of patients were placed in PP regardless of the severity of their ARDS [1, 2], compared to 30% during the pre-pandemic period [3]. Previous research has shown that early implementation of multiple 16-h sessions of PP in patients with moderate to severe ARDS was associated with reduced mortality, leading to updated French guidelines for managing ARDS patients [4, 5]. However, the duration of PP during the pandemic varied widely among patients, influenced by both patients’ response and the availability of trained ICU staff to turn patients to the supine position [6]. However, in a recent study, PP sessions lasting 24 h or more and implemented in patients with PaO_2_/FiO_2_ ratio greater than 150 mmHg were associated with increased survival in COVID-19 related ARDS [7]. It was therefore recently suggested that a longer duration of prone therapy may further improve ARDS patients’ outcome, but additional data with longer PP duration of PP are still awaited [8]. Moreover, PP increases the workload of ICU staff and is associated with certain risks, including pressure sores, potential hindrance to enteral nutrition and a higher rate of catheter-related blood stream infection [8]. Those risks potentially increase with the duration of each PP session. Consequently, the optimal duration of the PP in ARDS remains unknown.

The objective of this ancillary study was to assess the outcomes of intubated patients with COVID-19 related ARDS based on the cumulated duration of prone position during the first 48 h following ICU admission, using a large international cohort [2].

## Study design and methods

### Study design and patients

This study was an ancillary analysis of the ProneCOVID study using the COVID-ICU cohort [2, 9]. The ProneCOVID study was a previous analysis of a prospective, multicenter observational cohort study of 149 ICUs from 138 hospitals conducted across three European countries (France, Belgium and Switzerland) [9]. The ethical committees of Switzerland (BASEC #: 2020-00704), of the French Intensive Care Society (CE-SRLF 20-23) and of Belgium (2020-294) approved the previous study, and all patients or relatives had given their consent to be included in the COVID-ICU cohort.

The COVID-ICU cohort involved all consecutive patients ≥ 16 years old admitted to ICU from February 25, 2020, to May 4, 2020, with a recorded vital status at day 90, totaling 4643 patients. Laboratory confirmation for SARS-CoV-2 was defined as a positive result of real-time reverse transcriptase-polymerase chain reaction (RT-PCR) assay from either nasal or pharyngeal swabs, and/or lower respiratory tract aspirates. In the ProneCOVID study, a total of 2137 patients who were intubated and mechanically ventilated with a PaO_2_/FiO_2_ ratio < 300 mmHg and PEEP > 5 cmH_2_O, and no therapeutic limitations, within the first 24 h of ICU admission were included. This secondary analysis focused on the subgroup of intubated patients who were consecutively turned prone during the first 48 h after ICU admission. Patients who experienced PP only after 48 h, as well as patients for whom the time of proning was not reported were excluded. The study followed the STROBE statement for reporting observational studies [10].

Patients were categorized based on the cumulative duration of PP in hours during the first 48 h reflecting the total exposure to PP in hours from one or more sessions. Given that PP is now recommended for a 16-h daily duration for ARDS patients with PaO_2_/FiO_2_ ratio lower than 150 mmHg [4, 5], patients who were placed in PP for more than 32 h during this period of 48 h were assigned to the intensive PP group, while those who were placed in PP for 32 h or less were assigned to the standard PP group. The first 48 h after ICU admission were chosen in order to minimize the potential immortal time bias and to approximate an intention-to-treat approach similar to a randomized trial. Additionally, based on previous work, the management of ARDS during these initial 48 h period may be crucial for patients’ outcome. However, a second analysis including all patients experiencing PP during ICU stay was also performed and provided in Additional file 1. As previously described in COVID ICU cohort, decisions regarding indication for invasive mechanical ventilation and mechanical ventilation settings were left to the discretion of the clinicians at each participating centers. There was no information available regarding whether patients remained in PP continuously or were turned back to the supine position daily, only the duration of each individual session in the prone position was recorded.

### Data collection

Consistent with the previously published study [2], a standardized electronic case report form was completed each day at 10 a.m. by the study investigators. Baseline characteristics were collected at ICU admission: age, sex, body mass index (BMI), active smoking, Simplified Acute Physiology Score (SAPS) II score, Sequential Organ Failure Assessment (SOFA), treated hypertension, diabetes, long term corticosteroids, immunodeficiency, Clinical Frailty Scale, the date of the first symptom, and dates of the hospital and ICU admissions. All investigators were asked to provide the lowest arterial partial pressure of oxygen (PaO_2_) at Day-1 after intubation and the corresponding fraction of oxygen inspired (FiO_2_) to calculate PaO_2_/FiO_2_ ratio and to categorized severity ARDS according to the Berlin definition [11].

The initial 48-h period is defined as the time from 10 a.m. on the morning following admission to the ICU until 10 a.m. 48 h later (Additional data, Figure 1). Static compliance was defined by dividing the tidal volume by the driving pressure. The driving pressure was calculated by subtracting plateau pressure from positive end-expiratory pressure (PEEP). All biological data were collected at ICU admission. Proved concurrent bacterial pneumonia was defined by a positive bacterial culture at ICU admission in either a bronchoalveolar lavage sample, or in a blind protected specimen brush distal, or in endotracheal aspirates.

The primary outcome was mortality at day 60. Secondary outcomes included 28-day and 90-day mortality, ventilator free-days until day 28, need for extracorporeal membrane oxygenation (ECMO) and renal replacement therapy (RRT), use of neuromuscular blockers and inhaled nitric oxide. The ventilator-free days until day 28 was defined as the number of days alive and free from invasive mechanical ventilation for at least 48 consecutive hours. If the patient was re-intubated within 48 h of the extubation the variable was treated as zero ventilator-free days; if re-intubated after 48 h, the 48 h period was counted as ventilator-free days. Patients discharged from the ICU before 28 days were considered alive and free from invasive mechanical ventilation at 28 days. Nonsurvivors at day 28 were considered to have zero ventilator-free days [12]. The static compliance, the SOFA score and the PaO_2_/FiO_2_ ratio were also evaluated at day-3, day-5, day-7, day-14, day-21 and day-28.

### Statistical analysis

Characteristics of patients were described as counts and percentages for categorical variables, and as median and interquartile range (IQR) for quantitative variables. Categorical variables were compared using Chi-square or Fisher’s exact test, and quantitative variables were compared using Student’s t-test or Wilcoxon’s rank-sum test. Kaplan–Meier overall survival curves until day 60 were computed.

The primary endpoint was the 60-day mortality according to the prone therapy strategy during the first 48-h of ICU stay, i.e. a standard or an intensive therapy. To assess the prone therapy strategy effect on 60-day mortality, a target trial emulation framework was used, according to the cloning-censoring and weighting method. This method, which eliminates the risk of immortal time bias, has been well described elsewhere [13, 14].

At the beginning of the follow-up, all intubated patients could belong to either the intensive PP group (cumulative duration of PP > 32 h) or the standard PP group (cumulative duration of PP ≤ 32 h) at the end of day 2. Thus, firstly, we created two clones of each patient and assigned each clone to a different strategy: the intensive PP strategy, or standard PP strategy. This ensures that all patients were included in both “arms” of the emulated trial, regardless of their status at the end of 48 h (intensive PP or not). As a result, the baseline characteristics are identical in both “arms”, eliminating any baseline confounding. Secondly, we censored clones that were non-adherent to their assigned treatment strategy during follow-up (e.g., cumulated PP > 32 h at Day 2 for a clone included in the Standard “arm”, cumulated PP ≤ 32 h for a clone included in the Intensive “arm”). Thirdly, this informative censoring was addressed using inverse-probability-of-censoring weights (IPCW), in which uncensored observations are up-weighted to represent censored observations with similar characteristics. The probability of not being censored was estimated using a Cox model, with the following baseline variables: age (≥ 65 years vs. < 65 years), sex (female vs. male), admission period to intensive care unit (28 March vs. 29 March) (28 March being the median admission period), time between first symptoms and admission date (≥ 8 days vs. < 8 days) (8 being the median time), frailty scale (≥ 4 vs. < 4), body mass index (≥ 30 kg/m^2^ vs. < 30 kg/m^2^), arterial hypertension status (yes vs. no), diabetes status (yes vs. no), median SOFA score (≥ 11 vs. < 11), PaO_2_/FiO_2_ ratio (< 150 vs. ≥ 150 mmHg), static compliance (< 30 vs. ≥ 30), lymphopenia (lymphocytes < 1 vs. lymphocytes ≥ 1 G/L). Finally, the analysis of 60-day mortality using a Cox model weighted on IPCW was performed to estimate Hazard ratio, and its 95% confidence interval, associated to intensive PP and weighted Kaplan Meier curves. This analysis was performed on the complete cases data set, and a sensitivity analysis was performed using multiple imputations due to missing data, considering a maximum threshold of 30%. Multiple imputation was realized according to Vesin et al. [15], including all variables introduced in the analysis and the primary endpoint. The number of available data for each variable included in the analysis was provided in Additional file, Table 1. Proportional hazard assumption was assessed by inspecting the scaled Schoenfeld residuals and Harrel’s test [16]. We performed a subgroup analysis of mortality at day-60 according to PaO_2_/FiO_2_ ratio at day-1 (< or ≥ 150 mmHg), using the same approach in each subgroup, being aware of the limits for this analysis which remained exploratory.

All analyses were performed at a two-sided α level of 5% and conducted with R version 4.1.1 (R Foundation for Statistical Computing, Vienna, Austria).

## Results

### Characteristics of ICU intubated patients

**Fig. 1:**
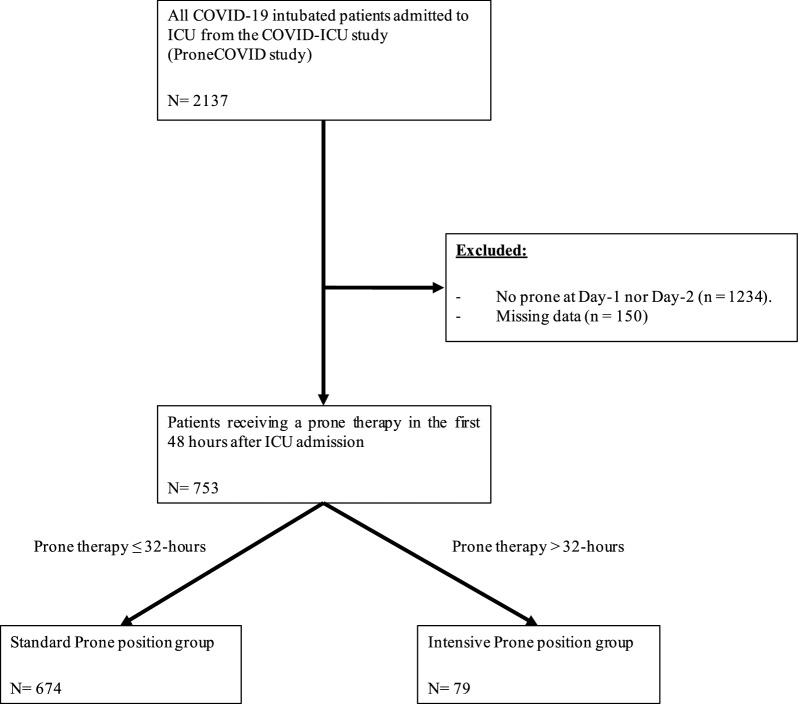
Study flow chart. *ICU* intensive care unit

ProneCOVID study enrolled 2137 intubated patients. In this secondary analysis, 753 patients met the inclusion criteria and were retrospectively analyzed, as shown in Fig. 1. The median age was 63 [55–70] years old. Approximatively half of patients were obese (BMI ≥ 30 kg/m^2^) and a treated for hypertension, and nearly one third had diabetes mellitus. Patients in the standard PP group were more likely to be men (76% vs. 58%, *p* = 0.001). All other characteristics at baseline were similar between the two groups and there were no group differences in severity of illness at admission using SOFA and SAPS II scores, or in the frailty clinical scale. Regarding ventilatory features, the median duration between ICU admission and the initiation of invasive mechanical ventilation was 2.3 [0.5–8.6] hours. The lowest PaO_2_/FiO_2_ ratio on Day-1 was 128 [88–174] mmHg and 37% of patients had a PaO_2_/FiO_2_ ratio of 150 mmHg or greater. Patients were ventilated with a median tidal volume of 6.1 [5.8–6.6] mL/kg and a median PEEP at 12 [10–14] cmH_2_O. All other baseline characteristics of patients are summarized in Table 1.

**Table 1:** Demographic, clinical and ventilatory characteristics of patients at admission

Variable	Standard prone position group (*n* = 674)	Intensive prone position group (*n* = 79)	All patients (*n* = 753)	*p*
Age (years), median [IQR]	64 [55; 70]	61 [54; 69]	63 [55; 70]	0.13
Sex, *n* (%)				
Men	506 (76%)	46 (58%)	552 (74%)	0.001
Women	163 (24%)	33 (42%)	196 (26%)	
Body mass index (kg/m^2^), median [IQR]	30 [26; 34]	31 [28; 35]	30 [27; 34]	0.02
≥ 30 kg/m^2^, *n* (%)	321 (51%)	48 (64%)	369 (52%)	0.03
Comorbidities, *n* (%)				
Active smokers	28 (4%)	0 (0%)	28 (4%)	0.06
Treated hypertension	348 (52%)	49 (62%)	397 (53%)	0.10
Known diabetes	216 (33%)	24 (30%)	240 (32%)	0.70
Immunodeficiency	45 (7%)	3 (4%)	48 (6%)	0.31
Long-term corticosteroids	20 (3%)	0 (0%)	20 (3%)	0.15
SAPS II score, median [IQR]	43 [33; 55]	43 [32; 52]	43 [32; 55]	0.80
SOFA score at ICU admission, median [IQR]	7 [4; 10]	8 [6; 11]	7 [4; 10]	0.18
Clinical frailty score ≤ 3, *n* (%)	539 (87%)	64 (83%)	603 (87%)	0.47
Time between first symptoms and ICU admission (days), median [IQR]	8 [6; 11.8]	8 [5; 11]	8 [6; 11]	0.65
Time between ICU admission and invasive mechanical ventilation (hours), median [IQR]	2.4 [0.5; 8.9]	1.8 [0.5; 3.8]	2.3 [0.5; 8.6]	0.25
Concomitant bacterial pneumonia, *n* (%)	62 (9%)	5 (6%)	67 (9%)	0.38
Patients receiving high-dose corticosteroids at Day-1, *n* (%)	67 (10%)	6 (8%)	73 (10%)	0.50
Invasive mechanical ventilation settings on Day-1, median [IQR]				
PaO_2_/FiO_2_ (mmHg)	126 [87; 172]	137 [96; 187]	128 [88; 174]	0.17
Tidal volume (mL)	410 [380; 446]	400 [364; 437]	410 [378; 444]	0.34
Tidal volume, mL/kg PBW	6 [5.8; 6.6]	6 [5.8; 6.7]	6 [5.8; 6.6]	0.70
Set PEEP (cmH_2_O)	12 [10; 14]	12 [10; 14]	12 [10; 14]	0.10
Plateau pressure (cmH_2_O)	25 [22; 28]	26 [24; 28]	25 [22; 28]	0.11
Driving pressure^a^ (cmH_2_O)	13 [11; 18]	14 [11; 19]	13 [11; 18]	0.41
Mechanical power^b^ (J/min)	28 [21; 36]	31 [21; 38]	29 [21; 36]	0.25
Ventilatory ratio^c^	1.9 [1.5; 2.3]	2 [1.6; 2.3]	1.9 [1.5; 2.3]	0.22
Static compliance^d^ (mL/cmH_2_O)	32 [26; 41]	32 [24; 41]	32 [26; 41]	0.48
Dynamic compliance^e^ (mL/cmH_2_O)	16 [13; 20]	15 [12; 20]	16 [13; 20]	0.36
Prone position support				
PP duration (hours) at Day 2, median [IQR]	14 [2; 16]	20 [17; 23]	15 [4.5; 17]	< 0.001
Cumulative duration of PP (hours) in the first 48 h, median [IQR]	16 [15; 21]	36 [34; 39]	17 [16; 24]	< 0.001
Cumulative duration of PP (hours) until Day 14, median [IQR]	48 [28; 81]	93 [56; 151]	52 [31; 88]	< 0.001
Blood gas on Day-1, median [IQR]				
pH	7.37 [7.30; 7.42]	7.35 [7.30; 7.39]	7.36 [7.30; 7.42]	0.20
PaCO_2_ (mmHg)	45 [38; 52]	46 [41; 52]	45 [39; 52]	0.13
PaO_2_/FiO_2_ (mmHg)	428 (64%)	47 (60%)	475 (63%)	0.49
< 150 mmHg, *n* (%)	25 [22; 28]	26 [23; 28]	25 [22; 28]	0.12
HCO_3_ (mmol/L)	7.4 [7.3; 7.4]	7.3 [7.3; 7.4]	7.4 [7.3; 7.4]	0.20
Lactate (mmol/L)	1.3 [1; 1.7]	1.3 [1; 1.4]	1.3 [1; 1.7]	0.53
Biology, median [IQR]				
Lymphocyte count (× 10^9^/L)	0.8 [0.5; 1.1]	0.8 [0.6; 1.1]	0.8 [0.5; 1.1]	0.38
Thrombocyte count (× 10^9^/L)	226 [170; 286]	225 [172; 287]	225 [170; 287]	0.97
Total bilirubin (µmol/L)	10 [7; 15]	10 [8; 14]	10 [7; 15]	0.94
Serum creatinine (µmol/L)	85 [65; 122]	88 [64; 125]	86 [65; 123]	0.71

IQR: interquartile range; SOFA: sequential organ failure assessment; SAPS II: simplified acute physiology score II; PaCO_2_: arterial partial pressure in carbon dioxide; PaO_2_: arterial partial pressure in oxygen; FiO_2_: fraction inspired in oxygen;

^a^Defined as plateau pressure—PEEP. If plateau pressure was missing, peak pressure was considered instead

^b^Mechanical power (J/min) = 0.098 × tidal volume × respiratory rate × (peak pressure − 1/2 × driving pressure). If not specified, peak pressure was considered equal to plateau pressure

^c^Defined as (minute ventilation × PaCO_2_) − (predicted bodyweight × 100 × 37.5)

^d^Normalized for ideal body weight. Defined as tidal volume/(Plateau pressure − PEEP)

^e^Normalized for ideal body weight. Defined as tidal volume/(Peak pressure − PEEP)

After the IPCW adjustment, results were analyzed in both complete case analysis including 354 patients and in multiple imputation analysis with all baseline population of 753 patients. In both weighted cohorts, there were no differences in key variables between prone therapy strategies (Tables 1 and 2).

**Table 2:** Primary and secondary outcomes

Outcome	Standard prone position group (*n* = 674)	Intensive prone position group (*n* = 79)	All patients (*n* = 753)	*p*
Primary outcome				
Mortality at 60-day, *n* (%)	261 (39%)	31 (39%)	292 (39%)	0.93
Secondary outcomes				
Mortality, *n* (%)				
At day 28	230 (34%)	28 (35%)	258 (34%)	0.82
At day 90	262 (39%)	31 (39%)	293 (39%)	0.95
Ventilator-free days until day 28, median [IQR]	2 [0; 14]	0.5 [0; 12]	1.5 [0; 14]	0.74
Extracorporeal membrane oxygenation, *n* (%)	68 (10%)	7 (9%)	75 (10%)	0.75
Inhaled nitric oxide, *n* (%)	161 (24%)	24 (30%)	185 (25%)	0.21
Neuromuscular blockers, *n* (%)	652 (97%)	74 (94%)	726 (96%)	0.19
Renal replacement therapy, *n* (%)	195 (29%)	22 (28%)	217 (29%)	0.83
Static compliance (mL/cmH_2_O), median [IQR]				
At Day-3	34 [25; 42]	29 [23; 36]	34 [25; 42]	0.03
At Day-5	32 [25; 40]	28 [21; 35]	31 [25; 40]	0.03
At Day-7	31 [24; 41]	29 [21; 37]	31 [23; 41]	0.11
SOFA score, median [IQR]				
At Day-7	10 [7; 12]	9 [7; 11]	10 [7; 12]	0.23
At Day-21	9 [7; 10]	5 [5; 12]	9 [5; 10]	–
At 28-day	7 [6; 10]	11 [11; 11]	7 [7; 11]	–
PaO_2_/FiO_2_ ratio (mmHg), median [IQR]				
At Day-3	151 [110; 198]	131 [105; 185]	148 [110; 195]	0.04
At Day-5	144 [105; 194]	134 [101; 209]	144 [105; 195]	0.54
At Day-7	149 [108; 189]	154 [108; 207]	150 [108; 192]	0.27

IQR: interquartile range; SOFA: sequential organ failure assessment; PaO_2_: arterial partial pressure in oxygen; FiO_2_: fraction inspired in oxygen; ICU: intensive care unit

### Prone position therapy

**Fig. 2:**
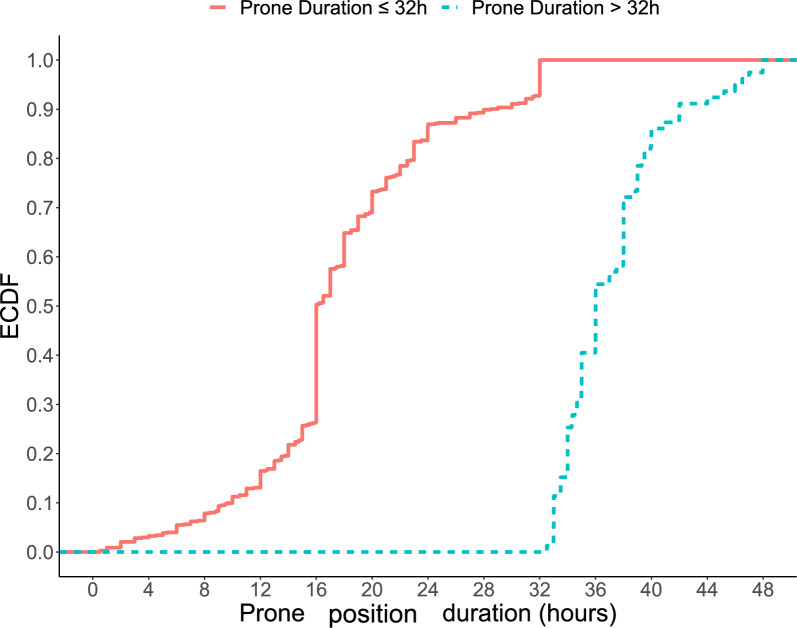
Empirical cumulative distribution function of the prone positioning duration during the first 48-h. *ECDF*:empirical cumulative distribution function

Among the 753 patients analyzed, 79 (10.5%) patients were classified in the intensive PP group with a median cumulative duration of PP of 36 [34–39] hours in the first 48 h. In the intensive group, no patients had a cumulative PP duration of 48 h, as illustrated in Fig. 2, and in Additional file, Figure 2. On the other hand, 89.5% of patients, corresponding to 674 patients, were classified in the standard PP group with a median cumulative duration of PP of 16 [15–21] hours. In this group, less than 10% of patients experienced a cumulative PP duration of 32 h (see Fig. 2, and Additional file, figure 2). Interestingly, the median duration of PP at Day 2 was 14 [2–16] hours in the standard PP group, while in the intensive group, patients had a median PP session of 20 [17–23] hours. Finally, the cumulative prone therapy duration during the first 14 days was longer in the intensive PP group than in the standard PP group (92 [56–151] vs. 48 [28–81] hours, *p* < 0.001). Characteristics of both groups at day-1 are summarized in Table 1.

### Outcomes

**Fig. 3:**
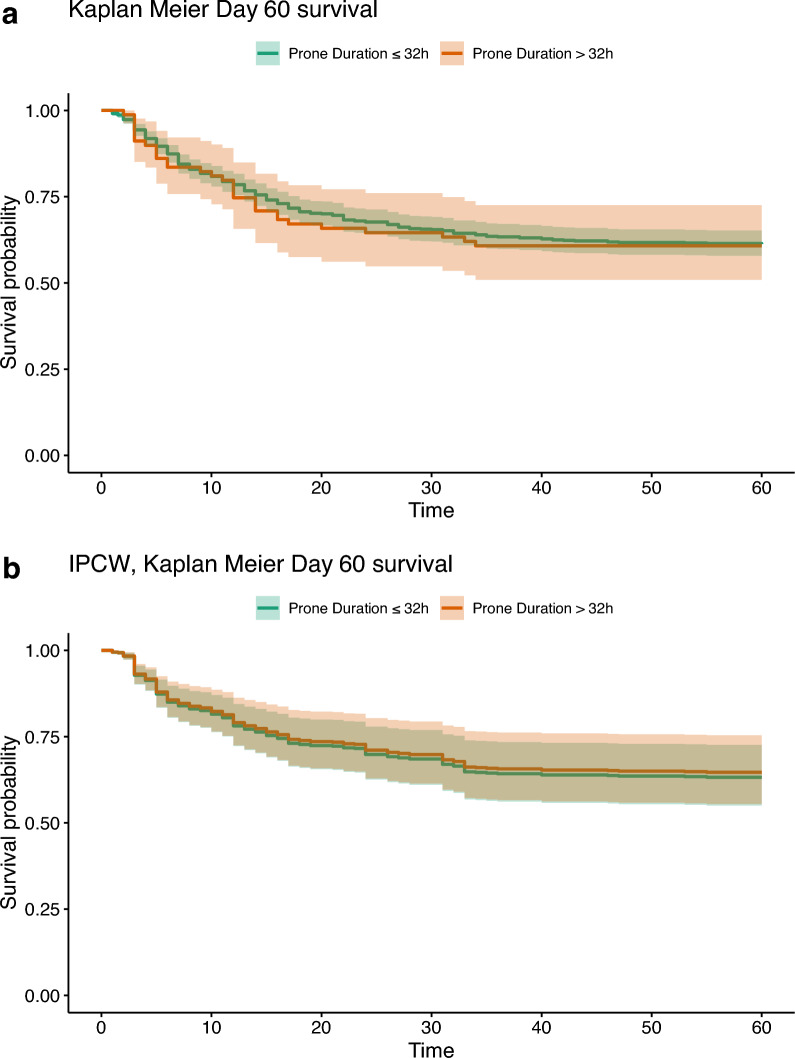
**a** Kaplan Meier curves according to prone positioning in the first 48-h after ICU admission before weighting adjustment in complete case population. **b** Kaplan Meier curves according to prone positioning in the first 48-h after ICU admission after weighting adjustment in complete case population. *ICU* intensive care unit

In the unadjusted analysis, mortality at day 28, 60, 90 were 34.3%, 38.8% and 38.9%, respectively. No significant difference in term of mortality at day 60 was observed (38.7% vs. 39.2%, *p* = 0.93), as reported in Table 2. The Kaplan–Meier survival curves for 60-day survival are shown in Fig. 3a.

**Fig. 4:**
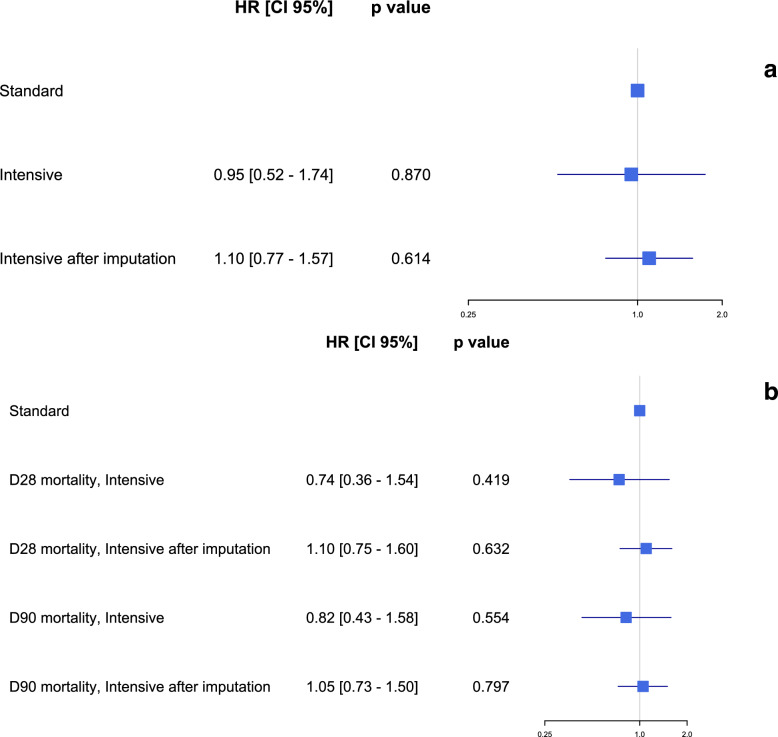
**a** Forest plot: hazard ratio of mortality at 60-day according to intensive prone positioning strategy in the first 48-h after ICU admission after weighting in baseline and complete case population. **b** Forest plot: hazard ratio of mortality at 28- and 90-day according to intensive prone positioning strategy in the first 48-h after ICU admission after weighting in baseline and complete case population. *ICU* intensive care unit, *HR* hazard ratio

After weighting, the IPCW weighted Cox models showed no significant difference in 60-day mortality in both analysis (hazard ratio (HR) 0.95 [0.52–1.74], *p* = 0.87 in complete case analysis, and 1.10 [0.77–1.57], *p* = 0.61 in multiple imputation analysis; Figs. 3b and 4a). Mortality at day 28 and day 90 were similar between the two study groups after both complete cases and multiple imputation IPCW weighted analysis (Fig. 4b). The number of ventilator-free days, the need for adjunctive therapies including inhaled nitric oxide, continuous neuromuscular blockers, ECMO and RRT were also similar in both the standard and the intensive PP groups (Table 2). During the first 28-days after ICU admission, there were no differences in the evolution of the PaO_2_/FiO_2_ ratio, the static compliance and the SOFA score between the standard and the intensive PP groups (Additional file, Figures 3, 4 and 5). An additional sensitivity analysis included PP, as continuous variable, in a multivariate Cox model did not find any significant association between the duration of PP and the mortality at day-60 and day-90, in line with our results, as shown in Table 2a–c in Additional file.

In subgroups of patients classified according to the PaO_2_/FiO_2_ ratio at Day-1 (< 150 mmHg or ≥ 150 mmHg), the 60-day mortality was 46.8% in patients receiving intensive prone therapy in comparison to 40.9% in patients receiving standard prone therapy in the subgroup of patients with a PaO_2_/FiO_2_ ratio less than 150 mmHg (Table 3, and Additional file, Figure 6). No association between the prone therapy strategy and survival at day 60 according to the baseline PaO_2_/FiO_2_ ratio was observed (HR 1.09 95% CI 0.56–2.15, p = 0.79 among patients with PaO_2_/FiO_2_ < 150 mmHg and HR 0.93 95% CI 0.27–3.16, p = 0.91 among patients with PaO_2_/FiO_2_ ≥ 150 mmHg) (Additional file, Table 3).

**Table 3:** Mortality according to the prone position strategy and the PaO_2_/FiO_2_ ratio

Variable	Prone position strategy according to PaO_2_/FiO_2_				
	< 150 mmHg (*n* = 475)		≥ 150 mmHg (*n* = 278)		*p*
	Standard (*n* = 428)	Intensive (*n* = 47)	Standard (*n* = 246)	Intensive (*n* = 32)	
60-day mortality, *n* (%)	175 (41%)	22 (47%)	86 (35%)	9 (28%)	0.16

PaO_2_: arterial partial pressure in oxygen; FiO_2_: fraction inspired in oxygen; ICU: intensive care unit

An additional analysis included all patients experiencing PP during ICU stay (n = 1987) was also provided in Additional file 1, and no significant difference in term of mortality at day 60 was observed between intensive PP group and standard PP group, as shown in Additional file 1, figure 6, figure 7 and Table 4.

## Discussion

In this secondary analysis of a multicenter, European cohort study, we found that a prolonged PP for more than 32 h during the first 48 h after ICU admission in patients intubated for COVID-19 related ARDS was not associated with a reduction in mortality at 60 days compared to shorter cumulative duration of PP. This finding remained consistent even in a subgroup analysis of patients with a PaO_2_/FiO_2_ ratio < 150 mmHg or a PaO_2_/FiO_2_ ratio ≥ 150 mmHg on Day-1.

Various strategies for PP duration have been proposed in previous studies. In a large clinical trial showing decreased mortality of PP, the PP group received 16 h of PP alternated with 8 h of supine positioning, with a median of 4 ± 4 sessions during their ICU stay. This approach resulted in a high exposure to prone therapy and showed a survival benefit [4]. However, during the COVID-19 pandemic one center reported increasing PP duration for a fixed amount of time, resulting in a median duration of 39 h [6]. Other centers maintained patients continuously in the prone position until clinical improvement was achieved [7, 16, 17, 18]. A retrospective multicenter trial showed that PP sessions, which were maintained for a median duration of 40 h but up to 10 days, was associated with reduced mortality compared to a fixed, 16-h duration [7]. All those protocols, which maintained patients continuously on the prone position and for a duration exceeding 24 h, are referred to under the name of prolonged PP. In this study, we compared the cumulative duration of PP over the first 2 days after ICU admission. However, we did not know whether this duration was obtained during a continuous session or whether it was the sum of two different PP sessions separated by time spent on the supine position, as described in large-scale clinical trial [4]. Thus, we referred to PP of a cumulated duration greater than 32 h over 2 days as “intensive PP”. We did not observe an association between intensive PP and increased survival compared to a cumulative duration of 32 h during the first 48 h. Our main hypothesis is that the difference in cumulative duration in the first 48 h between the two groups was not large enough to show to a significant impact on patient’s survival and that in some patients, the intensive PP was in fact the sum of two standard duration PP sessions. If future prospective randomized trials were to be conducted on PP sessions longer than 16 h, our findings support the testing of strictly greater than 24-h durations.

Similarly, the optimal timing of PP initiation remains uncertain. In a previous clinical study showing survival benefit of PP, PP was initiated within the first hour of randomization and after a first 12-h stabilization period in patients intubated for less than 36 h and requiring a FiO_2_ greater than 0.6 with a PaO_2_/FiO_2_ ratio inferior or equal to 150 mmHg [4]. However, PP is also a protective ventilation measure aimed at decreasing ventilator-induced lung injury by reducing ventral alveolar overdistention [19], and by homogenizing the strain to lung tissue associated with mechanical ventilation on inflamed alveoli [20–22]. Studies have shown that PP improves blood oxygenation by homogenizing the distribution of pulmonary ventilation/perfusion ratio. However, it is important to note that improvement in gas exchange is not sufficient to predict an improvement in survival in cases of ARDS unrelated to COVID-19 [19, 22–26]. It also preserves systemic hemodynamics [27], particularly right ventricular function [28]. Therefore, the criteria of a PaO_2_/FiO_2_ ratio less than or equal to 150 mmHg was likely chosen to select severe patients, in order to demonstrate an impact on mortality. Physiologically, PP may be beneficial for patients with less severe hypoxemia by preventing clinical deterioration [2, 29]. Our study indicates that the implementation of PP in less severe cases of ARDS (with a PaO_2_/FiO_2_ ratio > 150 mmHg) may have a greater impact than in more severe cases. This hypothesis is supported by the high mortality rate observed in patients with a PaO_2_/FiO_2_ ratio > 150 mmHg in the Lung Safe study, which showed that a large proportion of patients with ARDS worsened in the first week, and that it might therefore be appropriate to start PP earlier, making them optimal candidates for the protective impact of PP [30]. Although no conclusions can be drawn from this subgroup analysis of our study, our hypothesis is that implementing PP when ARDS is not yet too severe and the lung parenchyma is not yet too impacted by both the disease and ventilatory-induced lung injuries (VILI) might be more effective than implementing PP in patients in which the extend of lung injuries is more important.

Surprisingly, in the more severe group of patients with a PaO_2_/FiO_2_ ratio < 150 mmHg, the intensive PP group had a slightly lower survival rate, although this difference was not statistically significant. Several hypotheses could explain this finding. Firstly, as mentioned above, it is unclear whether the reported PP duration was obtained during a single continuous session or whether it was a cumulative duration with separate PP sessions and periods of supine positioning. The slightly higher mortality observed in the intensive group may be explained by the confounding variable of uncertainty. Another hypothesis is that this result may be due to sampling fluctuation. Additionally, it is possible that the physiopathology observed in the ARDS related to COVID-19 may be different from other ARDS causes. Compared to *Influenza* infection, the ventilation/perfusion ratio impairment is likely due to higher prevalence of microthrombi in the pulmonary circulation, resulting in an increased dead-space effect [31]. Therefore, the benefit of prone therapy in COVID-19 may be more related to the redistribution of pulmonary perfusion, which improves the ventilation perfusion ratio, a transitory effect, rather than alveolar recruitment, a more durable effect [32].

Our study has several strengths. Firstly, it has a large sample size, including patients from 149 different centers across 3 countries, all within included over a short period of time. Secondly, our study focuses on early and daily PP in the first 48 h after ICU admission, which is similar to the approach taken in the recent large-scale clinical trial [4]. This allows for the evaluation of homogeneous patients of similar severity requiring daily PP, thereby the risk of cofounding factors. However, according to the previous report, most patients were placed in the prone position by day 3, and some only received one prone positioning session within the first 48 h, explaining the short median cumulative PP duration in the standard group during the first 48 h [2]. As a result, many patients were excluded based on our inclusion criteria. Another limitation of this study is its retrospective design. Although a robust statistical analysis method was used, some unmeasured confounders may have contributed to the lack of statistically significant differences between the two groups. Examples of these include the variation in limitation of life-sustaining therapies between centres in the COVID-ICU cohort [33], and the potential role of organizational factors such as bed availability in the decision-making process for proning patients [34]. Furthermore, it was unclear whether the cumulative duration of PP reflected a single session or multiple sessions. It is currently unclear whether the repeated long sessions of prone therapy are more beneficial than other protocols for the same cumulative duration of prone positioning. Additionally, we did not report any complications related to prone positioning, such as pressure sores or facial oedema.

## Interpretation

An intensive prone therapy strategy, defined as cumulative duration of PP of more than 32 h during the first 48 h of ICU admission, in intubated patients with ARDS related to COVID-19 was not associated with a lower mortality at 60 days compared to a standard prone therapy strategy.

## Supplementary Information

Additional file 1. Additional information about the baseline characteristics and the statistical analysis. Additional Tables and Figures. Figure 1. Timeline and study period considered after ICU admission. Table 1. Proportion of missing data for each variable included in the analysis. Figure 2. Distribution of cumulative duration of prone positioning during the first 48 h after ICU admission. Figure 3. Evolution of the P_a_O_2_/F_i_O_2_ ratio during the first 28-days according to the prone strategy. Figure 4. Evolution of the static compliance during the first 28-days according to the prone strategy. Figure 5. Evolution of the SOFA score during the first 28-days according to the prone strategy. Table 2a. Estimated hazard ratio from a multivariate Cox model including day-60 survival associated with multiple variables in both the multiple imputation and complete case populations. b. Estimated hazard ratio from a multivariate Cox model including day-28 survival associated with multiple variables in both the multiple imputation and complete case populations. c. Estimated hazard ratio from a multivariate Cox model including day-90 survival associated with multiple variables in both the multiple imputation and complete case populations. Table 3. Estimated hazard ratio of the day-60 survival associated with the prone therapy strategy according to the prone position therapy strategy and the PaO_2_/FiO_2_ ratio at ICU admission before and after weighting in both multiple imputation and complete case population. Figure 6. Flow chart study included all ICU patients experiencing prone therapy during ICU stay. Table 4. Estimated hazard ratio of the day-60 survival associated with the prone therapy strategy including all patients experiencing prone therapy during ICU stay before and after weighting in both multiple imputation and complete case population. Figure 7a. Kaplan Meier curves according to prone therapy strategy including all patients experiencing prone therapy during ICU stay before weighting adjustment in complete case population. b. Kaplan Meier curves according to prone therapy strategy including all patients experiencing prone therapy during ICU stay after weighting adjustment in complete case population.

## Data Availability

The datasets used and/or analyzed during the current study are available from the corresponding author on reasonable request.

## Abbreviations

COVID-19: Coronavirus disease 2019
PP: Prone position
ICU: Intensive care unit
ARDS: Acute respiratory distress syndrome
RT-PCR: Real-time reverse transcriptase-polymerase chain reaction
BMI: Body mass index
SOFA: Sequential Organ Failure Assessment
PaO_2_: Arterial partial pressure of oxygen
FiO_2_: Fraction inspired of oxygen
SAPS II: Simplified Acute Physiology Score II
PEEP: Positive end-expiratory pressure
ECMO: Extracorporeal membrane oxygenation
IPTW: Inverse probability of treatment weighting
PS: Propensity score
