# Correction to: Impact of intensive prone position therapy on outcomes in intubated patients with ARDS related to COVID-19

**DOI:** 10.1186/s13613-024-01374-3

**Published:** 2024-09-11

**Authors:** Christophe Le Terrier, Thaïs Walter, Said Lebbah, David Hajage, Florian Sigaud, Claude Guérin, Luc Desmedt, Steve Primmaz, Vincent Joussellin, Chiara Della Badia, Jean Damien Ricard, Jérôme Pugin, Nicolas Terzi, COVID-ICU Group on behalf of the REVA Network and the COVID-ICU Investigators

**Affiliations:** 1 Division of Intensive Care, Faculty of Medicine, Geneva University Hospitals and The University of Geneva, Gabrielle‑Perret‑Gentil 4, Geneva, 1205 Switzerland; 2 Division of Intensive Care, Saint-Louis Hospital, Greater Paris Hospital, Paris, France; 3 Département de Santé Publique, Centre de Pharmaco‑épidémiologie, AP-HP, Paris, France; 4 Division of Intensive Care, Grenoble Alpes University Hospital, Grenoble, France; 5 Division of Intensive Care, Edouard Herriot University Hospital, Lyon, France; 6 Medical Intensive Care Unit, Nantes Hôtel-Dieu University Hospital, Nantes, France; 7 Medical Intensive Care Unit, University Hospital of Rennes, Rennes, France; 8 UMR1137 IAME, INSERM, Université Paris Cité, Paris, 75018 France; 9 DMU ESPRIT, Service de Médecine Intensive Réanimation, Université Paris Cité, AP-HP, Hôpital Louis Mourier, Colombes, 92700 France

**Correction to: Annals of Intensive Care (2024) 14:100.**

10.1186/s13613-024-01340-z.

In this article the author name Joussellin was incorrectly written as Jousselin.

The original article has been corrected.

