# Supplementary material for: Impact of intensive prone position therapy on outcomes in intubated patients with ARDS related to COVID-19
Source: Ann Intensive Care. 2024 Jun 27;14:100. doi: 10.1186/s13613-024-01340-z (PMC11211313; doi:10.1186/s13613-024-01340-z)

**ADDITIONAL FILE**

**Impact of intensive prone position therapy on outcomes in intubated patients with ARDS related to COVID-19**

Christophe Le Terrier, MD^1^; Thaïs Walter, MD^2^; Said Lebbah, MD^3^; David Hajage, MD^3^; Florian Sigaud, MD^4^; Claude Guérin, MD^5^; Luc Desmedt, MD^6^; Steve Primmaz, MD^1^; Vincent Jousselin, MD^7^; Chiara Della Badia, MD^1^; Jean-Damien Ricard, MD^7, 8^; Jérôme Pugin, MD^1^; Nicolas Terzi, MD^9^; COVID-ICU Group on behalf of the REVA Network and the COVID-ICU Investigators*

* COVID-ICU Investigators are listed at the end of the manuscript.

**^1^** Division of Intensive care, Geneva University Hospitals and the University of Geneva Faculty of Medicine, Geneva, Switzerland

**^2^** Division of Intensive Care, Saint-Louis Hospital, Greater Paris Hospital, France

**^3^** AP-HP, Département de Santé Publique, Centre de Pharmaco-épidémiologie, Paris, France

**^4^** Division of Intensive Care, Grenoble Alpes University Hospital, Grenoble, France

**^5^** Division of Intensive Care, Edouard Herriot University Hospital, Lyon, France

**^6^** Medical Intensive Care unit, Nantes Hôtel-Dieu University Hospital, Nantes, France

**^7^** Medical Intensive Care unit, University Hospital of Rennes, Rennes, France

^8^ Université Paris Cité, UMR1137 IAME, INSERM, 75018, Paris, France.

^9^ Université Paris Cité, AP-HP, Hôpital Louis Mourier, DMU ESPRIT, Service de Médecine Intensive Réanimation, 92700, Colombes, France

**Keywords:** Acute respiratory distress syndrome – Intubation – COVID-19 – Mortality – Intensive Prone position– Intensive care unit.

**Running title**: *Intensive Prone positioning in ARDS due to COVID-19.*

**Submitted to:** *Annals of Intensive care*

**Word count**: Abstract 349.; Manuscript 3852; 3 Tables; 4 Figures; 1 Additional file.

**Corresponding author**

Christophe Le Terrier, MD

Division of Intensive care

University Hospitals of Geneva

Gabrielle-Perret-Gentil 4

1205 Genève, Switzerland

E-mail: Christophe.leterrier@hcuge.ch

**COVID-ICU collaborative group**

Investigators are listed at the end of the manuscript

E-mail covid.icu@gmail.com

**Contents**

**Additional Tables and Figures**

**Figure 1** Timeline and study period considered after ICU admission 4

**Table 1** Proportion of missing data for each variable included in the analysis 5

**Figure 2** Distribution of cumulative duration of prone positioning during the first 48 hours after ICU admission 6

**Figure 3** Evolution of the PaO_2_/FiO_2_ ratio during the first 28-days according to the prone strategy 7

**Figure 4** Evolution of the static compliance during the first 28-days according to the prone strategy 8

**Figure 5** Evolution of the SOFA score during the first 28-days according to the prone strategy 9

**Table 2 a.** Estimated hazard ratio from a multivariate Cox model including day-60 survival associated with multiple variables in both the multiple imputation and complete case populations. **b.** Estimated hazard ratio from a multivariate Cox model including day-28 survival associated with multiple variables in both the multiple imputation and complete case populations. **c.** Estimated hazard ratio from a multivariate Cox model including day-90 survival associated with multiple variables in both the multiple imputation and complete case populations 10

**Table 3** Estimated hazard ratio of the day-60 survival associated with the prone therapy strategy according to the prone position therapy strategy and the PaO_2_/FiO_2_ ratio at ICU admission before and after weighting in both multiple imputation and complete case population 13

**Figure 6** Flow chart study included all ICU patients experiencing prone therapy during ICU stay. 14

**Table 4** Estimated hazard ratio of the day-60 survival associated with the prone therapy strategy including all patients experiencing prone therapy during ICU stay before and after weighting in both multiple imputation and complete case population 15

**Figure 7 a.** Kaplan Meier curves according to prone therapy strategy including all patients experiencing prone therapy during ICU stay before weighting adjustment in complete case population. **b.** Kaplan Meier curves according to prone therapy strategy including all patients experiencing prone therapy during ICU stay after weighting adjustment in complete case population. 16

**Figure 1.** Timeline and study period considered after ICU admission

**Table 1** Proportion of missing data for each variable included in the analysis

| **Variable** | **n** | **Missing (n)** | **Missing (%)** |
| --- | --- | --- | --- |
| Prone position duration, Hours | 753 | 0 | 0.0 |
| Age | 753 | 0 | 0.0 |
| Sex | 748 | 5 | 0.7 |
| BMI | 705 | 48 | 6.4 |
| Hypertension | 745 | 8 | 1.1 |
| Diabetes | 743 | 10 | 1.3 |
| Frailty scale | 696 | 57 | 7.6 |
| SOFA score | 647 | 106 | 14.1 |
| PaO2/FiO2 ratio | 753 | 0 | 0.0 |
| Static compliance | 548 | 205 | 27.2 |
| Lymphopenia | 638 | 115 | 15.3 |
| Admission period to intensive care unit | 753 | 0 | 0.0 |
| Time between first symptoms and admission date | 718 | 35 | 4.6 |

**Figure 2** Distribution of cumulative duration of prone positioning during the first 48 hours after ICU admission

Prone therapy duration (hours)

**Figure 3** Evolution of the PaO_2_/FiO_2_ ratio during the first 28-days according to the prone strategy

**Figure 4** Evolution of the static compliance during the first 28-days according to the prone strategy


**Figure 5** Evolution of the SOFA score during the first 28-days according to the prone strategy

|  | **Multiple imputation analysis (N = 753)** | | **Complete case analysis (N = 354)** | |
| --- | --- | --- | --- | --- |
| **Variable** | **HR [CI 95%]** | **p** | **HR [CI 95%]** | **p** |
| Prone position duration (continuous variable), Hours | 1.02 [0.99 - 1.04] | 0.17 | 1.01 [0.99 - 1.02] | 0.24 |
| Age ≥ 65 years | 1.91 [1.32 - 2.77] | <0.001 | 2.03 [1.58 - 2.61] | <0.001 |
| Female | 0.65 [0.41 - 1.02] | 0.06 | 0.77 [0.57 - 1.04] | 0.08 |
| BMI ≥ 30kg/m² | 0.77 [0.53 - 1.1] | 0.15 | 0.8 [0.62 - 1.03] | 0.09 |
| Hypertension | 0.89 [0.6 - 1.31] | 0.55 | 0.95 [0.73 - 1.23] | 0.70 |
| Diabetes | 1.61 [1.1 - 2.34] | 0.01 | 1.45 [1.12 - 1.88] | 0.005 |
| Frailty scale ≥ 4 | 1.82 [1.13 - 2.92] | 0.01 | 1.67 [1.22 - 2.29] | 0.001 |
| SOFA score ≥ 11 | 2.21 [1.51 - 3.24] | <0.001 | 2.15 [1.65 - 2.81] | <0.001 |
| PaO2/FiO2 ratio < 150 mmHg | 1.26 [0.86 - 1.86] | 0.24 | 1.25 [0.97 - 1.6] | 0.09 |
| Static compliance < 30 | 2.26 [1.54 - 3.3] | <0.001 | 1.64 [1.22 - 2.2] | 0.002 |
| Lymphopenia, lymphocytes < 1 G/L | 1.23 [0.84 - 1.78] | 0.29 | 1.01 [0.78 - 1.31] | 0.95 |
| Admission period to intensive care unit, ≤ 28 March | 0.86 [0.6 - 1.23] | 0.40 | 0.91 [0.71 - 1.16] | 0.45 |
| Time between first symptoms and admission date, ≥ 8 days | 0.66 [0.46 - 0.94] | 0.02 | 0.73 [0.57 - 0.94] | 0.02 |

**Table 2 a.** Estimated hazard ratio from a multivariate Cox model including day-60 survival associated with multiple variables in both the multiple imputation and complete case populations.

|  | **Multiple imputation analysis (N = 753)** | | **Complete case analysis (N = 354)** | |
| --- | --- | --- | --- | --- |
| **Term** | **HR [CI 95%]** | **p** | **HR [CI 95%]** | **p** |
| Prone position duration (continuous variable), Hours | 1.02 [1.00 - 1.05] | 0.046 | 1.01 [1.00 - 1.03] | 0.11 |
| Age ≥ 65 years | 1.93 [1.31 - 2.86] | <0.001 | 1.98 [1.50 - 2.62] | <0.001 |
| Female | 0.58 [0.36 - 0.94] | 0.029 | 0.75 [0.53 - 1.05] | 0.10 |
| BMI ≥ 30kg/m² | 0.78 [0.53 - 1.15] | 0.22 | 0.83 [0.63 - 1.08] | 0.17 |
| Hypertension | 0.86 [0.57 - 1.29] | 0.46 | 0.95 [0.72 - 1.25] | 0.72 |
| Diabetes | 1.62 [1.09 - 2.43] | 0.018 | 1.33 [1.00 - 1.76] | 0.048 |
| Frailty scale ≥ 4 | 2.00 [1.22 - 3.28] | 0.006 | 1.87 [1.34 - 2.60] | <0.001 |
| SOFA score ≥ 11 | 2.18 [1.46 - 3.26] | <0.001 | 2.13 [1.59 - 2.85] | <0.001 |
| PaO2/FiO2 ratio < 150 mmHg | 1.35 [0.89 - 2.05] | 0.16 | 1.24 [0.94 - 1.63] | 0.12 |
| Static compliance < 30 | 2.51 [1.68 - 3.75] | <0.001 | 1.77 [1.12 - 2.81] | 0.028 |
| Lymphopenia, lymphocytes < 1 G/L | 1.14 [0.77 - 1.69] | 0.52 | 0.97 [0.72 - 1.32] | 0.87 |
| Admission period to intensive care unit, ≤ 28 March | 0.83 [0.57 - 1.22] | 0.35 | 0.91 [0.70 - 1.19] | 0.51 |
| Time between first symptoms and admission date, ≥ 8 days | 0.74 [0.51 - 1.08] | 0.12 | 0.72 [0.55 - 0.93] | 0.012 |

**b.** Estimated hazard ratio from a multivariate Cox model including day-28 survival associated with multiple variables in both the multiple imputation and complete case populations.

**c.** Estimated hazard ratio from a multivariate Cox model including day-90 survival associated with multiple variables in both the multiple imputation and complete case populations**.**

|  | **Multiple imputation analysis (N = 753)** | | **Complete case analysis  (N = 354)** | |
| --- | --- | --- | --- | --- |
| **Term** | **HR [CI 95%]** | **p** | **HR [CI 95%]** | **p** |
| Prone position duration (continuous variable), Hours | 1.02 [0.99 - 1.04] | 0.16 | 1.01 [0.99 - 1.02] | 0.34 |
| Age ≥ 65 years | 1.92 [1.33 - 2.77] | <0.001 | 2.01 [1.55 - 2.61] | <0.001 |
| Female | 0.65 [0.41 - 1.02] | 0.059 | 0.76 [0.55 - 1.05] | 0.10 |
| BMI ≥ 30kg/m² | 0.77 [0.53 - 1.10] | 0.15 | 0.78 [0.60 - 1.01] | 0.064 |
| Hypertension | 0.89 [0.60 - 1.31] | 0.56 | 0.96 [0.74 - 1.25] | 0.79 |
| Diabetes | 1.61 [1.11 - 2.35] | 0.013 | 1.42 [1.10 - 1.85] | 0.009 |
| Frailty scale ≥ 4 | 1.81 [1.13 - 2.91] | 0.014 | 1.73 [1.26 - 2.36] | <0.001 |
| SOFA score ≥ 11 | 2.22 [1.51 - 3.24] | <0.001 | 2.16 [1.64 - 2.84] | <0.001 |
| PaO2/FiO2 ratio < 150 mmHg | 1.26 [0.86 - 1.85] | 0.24 | 1.23 [0.95 - 1.58] | 0.12 |
| Static compliance < 30 | 2.26 [1.55 - 3.30] | <0.001 | 1.62 [1.05 - 2.49] | 0.043 |
| Lymphopenia, lymphocytes < 1 G/L | 1.23 [0.84 - 1.79] | 0.29 | 0.99 [0.75 - 1.31] | 0.94 |
| Admission period to intensive care unit, ≤ 28 March | 0.86 [0.60 - 1.23] | 0.40 | 0.90 [0.70 - 1.15] | 0.38 |
| Time between first symptoms and admission date, ≥ 8 days | 0.66 [0.46 - 0.94] | 0.021 | 0.73 [0.58 - 0.94] | 0.013 |

**Table 3** Estimated hazard ratio of the day-60 survival associated with the prone therapy strategy according to the prone position therapy strategy and the PaO_2_/FiO_2_ ratio at ICU admission before and after weighting in both multiple imputation and complete case population

|  | **Variable** | **HR** | **CI 95%** | **p** |
| --- | --- | --- | --- | --- |
| PaO_2_/FiO_2_ ≥ 150 | Intensive, Complete case analysis, n = 134 | 0.93 | [0.27 - 3.16] | 0.90 |
|  | Intensive, Imputation population analysis, n = 278 | 0.82 | [0.41 - 1.63] | 0.57 |
| PaO_2_/FiO_2_ < 150 | Intensive, Complete case analysis, n = 220 | 1.09 | [0.56 - 2.15] | 0.79 |
|  | Intensive, Imputation population analysis, n = 475 | 1.21 | [0.79 - 1.87] | 0.38 |

**Figure 6.** Flow chart study for a second analysis considering all ICU patients experiencing prone therapy during ICU stay.


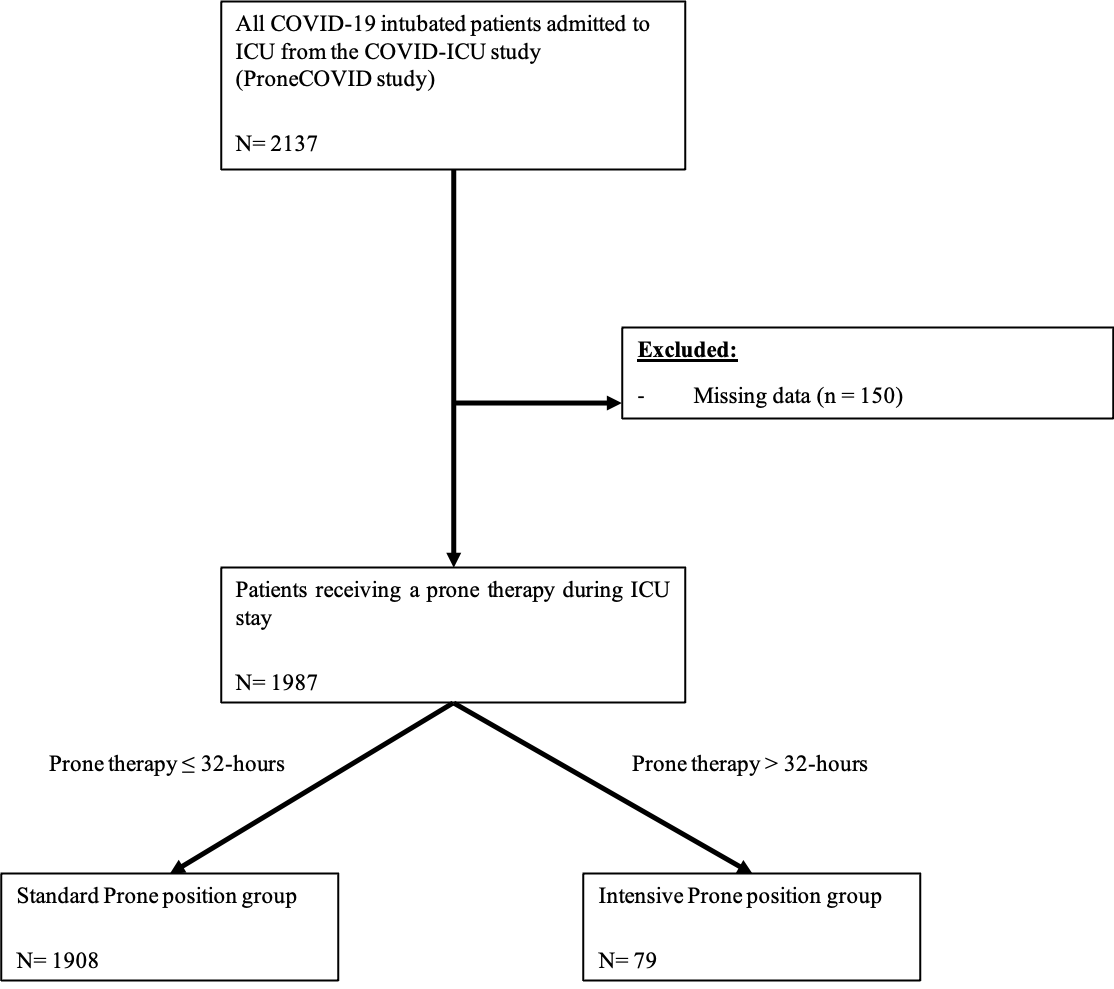


**Table 4** Estimated hazard ratio of the days 60, 28 and 90 mortalities associated with the prone therapy strategy for a second analysis considering all patients experiencing prone therapy during ICU stay before and after weighting in both multiple imputation and complete case population

| **Variable** | **HR** | **CI 95%** | **p** |
| --- | --- | --- | --- |
| Intensive, Complete case analysis | 1.15 | [0.59 – 2.22] | 0.687 |
| Intensive, Imputation population analysis, n =1908 | 1.30 | [0.94 - 1.79] | 0.116 |

**Figure 7 a.** Kaplan Meier curves according to prone therapy strategy including all patients experiencing prone therapy during ICU stay before weighting adjustment in complete case population. **b.** Kaplan Meier curves according to prone therapy strategy including all patients experiencing prone therapy during ICU stay after weighting adjustment in complete case population.


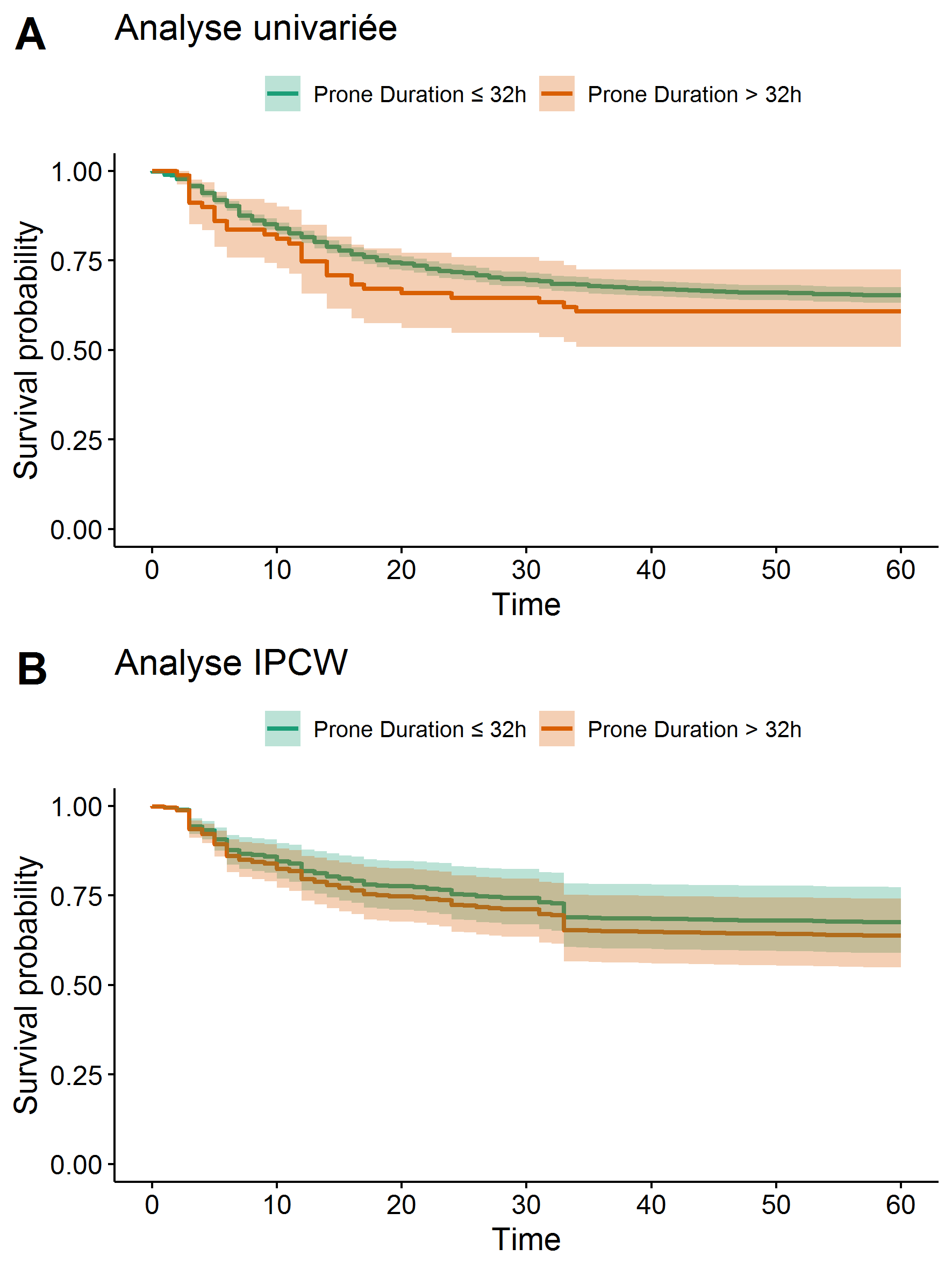

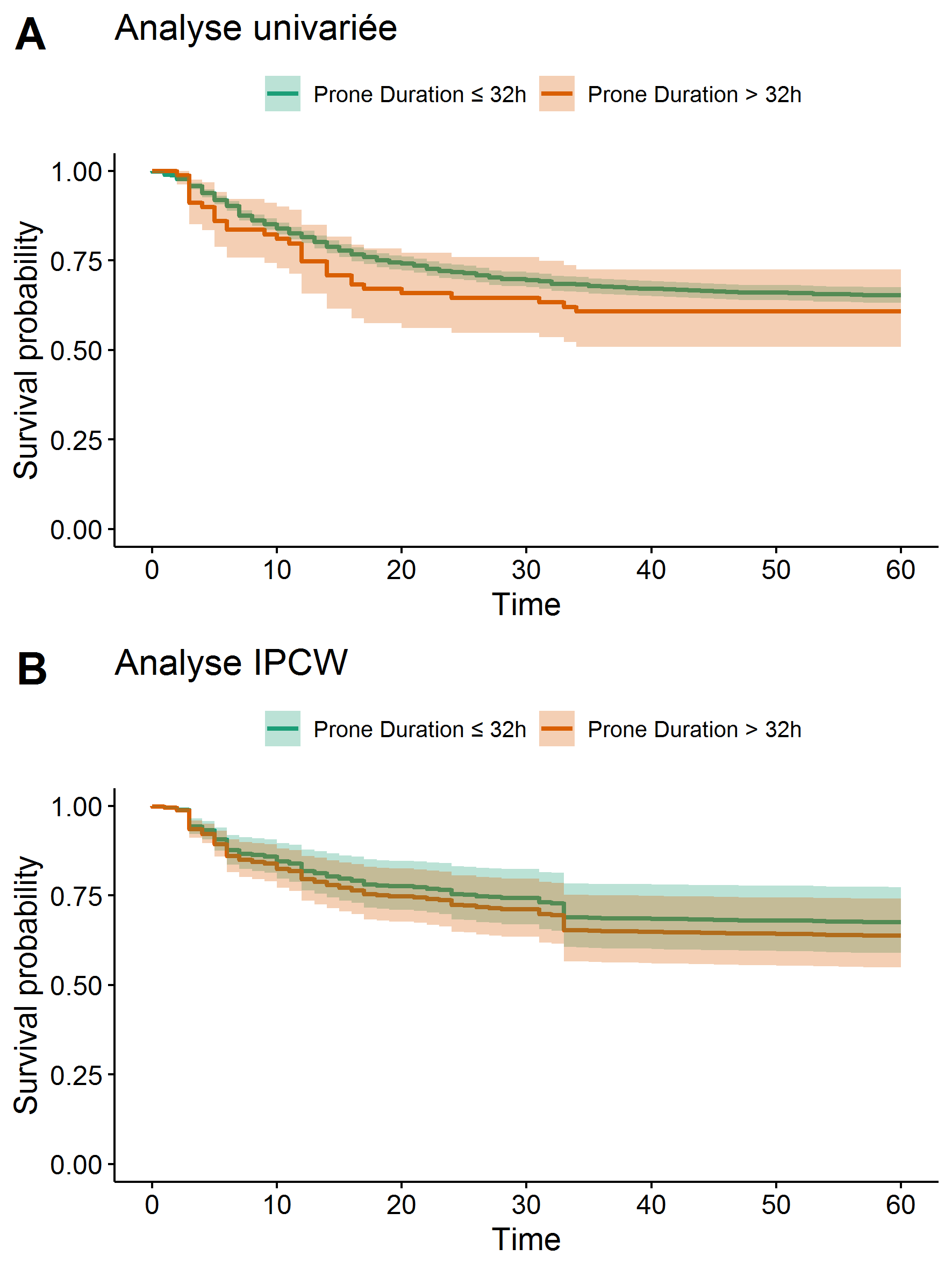

Supplement: Supplementary file 1 — Additional file 1. Additional information about the baseline characteristics and the statistical analysis. Additional Tables and Figures. Figure 1. Timeline and study period considered after ICU admission. Table 1. Proportion of missing data for each variable included in the analysis. Figure 2. Distribution of cumulative duration of prone positioning during the first 48 h after ICU admission. Figure 3. Evolution of the PaO2/FiO2 ratio during the first 28-days according to the prone strategy. Figure 4. Evolution of the static compliance during the first 28-days according to the prone strategy. Figure 5. Evolution of the SOFA score during the first 28-days according to the prone strategy. Table 2a. Estimated hazard ratio from a multivariate Cox model including day-60 survival associated with multiple variables in both the multiple imputation and complete case populations. b. Estimated hazard ratio from a multivariate Cox model including day-28 survival associated with multiple variables in both the multiple imputation and complete case populations. c. Estimated hazard ratio from a multivariate Cox model including day-90 survival associated with multiple variables in both the multiple imputation and complete case populations. Table 3. Estimated hazard ratio of the day-60 survival associated with the prone therapy strategy according to the prone position therapy strategy and the PaO2/FiO2 ratio at ICU admission before and after weighting in both multiple imputation and complete case population. Figure 6. Flow chart study included all ICU patients experiencing prone therapy during ICU stay. Table 4. Estimated hazard ratio of the day-60 survival associated with the prone therapy strategy including all patients experiencing prone therapy during ICU stay before and after weighting in both multiple imputation and complete case population. Figure 7a. Kaplan Meier curves according to prone therapy strategy including all patients experiencing prone therapy during ICU stay befor [file 13613_2024_1340_MOESM1_ESM.docx]
